# The DOCK protein family in vascular development and disease

**DOI:** 10.1007/s10456-021-09768-8

**Published:** 2021-02-06

**Authors:** Clare E. Benson, Laura Southgate

**Affiliations:** 1 Genetics Research Centre, Molecular and Clinical Sciences Research Institute, St. George’s University of London, Cranmer Terrace, London, SW17 0RE UK; 2 Department of Medical & Molecular Genetics, Faculty of Life Sciences & Medicine, King’s College London, London, SE1 9RT UK

**Keywords:** Angiogenesis, Cdc42, Dock, Rac1, Vascular disease, Vasculogenesis

## Abstract

The vascular network is established and maintained through the processes of vasculogenesis and angiogenesis, which are tightly regulated during embryonic and postnatal life. The formation of a functional vasculature requires critical cellular mechanisms, such as cell migration, proliferation and adhesion, which are dependent on the activity of small Rho GTPases, controlled in part by the dedicator of cytokinesis (DOCK) protein family. Whilst the majority of DOCK proteins are associated with neuronal development, a growing body of evidence has indicated that members of the DOCK family may have key functions in the control of vasculogenic and angiogenic processes. This is supported by the involvement of several angiogenic signalling pathways, including chemokine receptor type 4 (CXCR4), vascular endothelial growth factor (VEGF) and phosphatidylinositol 3-kinase (PI3K), in the regulation of specific DOCK proteins. This review summarises recent progress in understanding the respective roles of DOCK family proteins during vascular development. We focus on existing in vivo and in vitro models and known human disease phenotypes and highlight potential mechanisms of DOCK protein dysfunction in the pathogenesis of vascular disease.

## Introduction

**Fig. 1:**
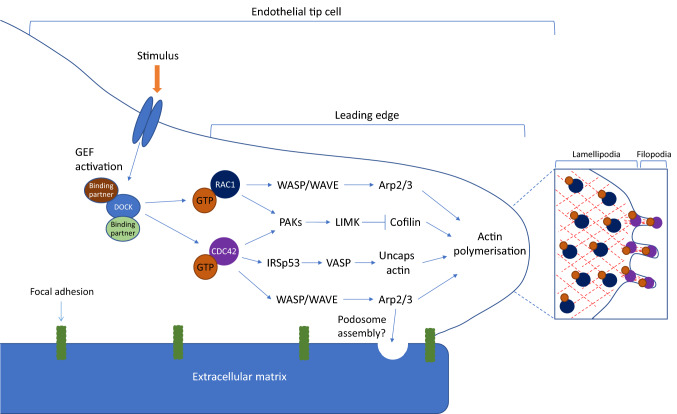
DOCK protein activation of small Rho GTPases in endothelial tip cells. Schematic of the effector pathways downstream of RAC1 and CDC42, following GEF-mediated activation by DOCK proteins. The specific DOCK subfamily binding partners are detailed in Fig. 3. Activation of RAC1 and CDC42 regulates lamellipodia or filopodia formation, respectively, via actin polymerisation and cytoskeleton organisation. CDC42 is also involved in podosome assembly, however the regulation of this process by DOCK proteins remains unknown. *Arp2/3* actin-related protein complex 2/3, *GDP* guanosine-5′-diphosphate, *GTP* guanosine-5′-triphosphate, *GEF* guanine nucleotide exchange factor, *LIMK* LIM kinase, *PAK* serine/threonine-protein kinase; *VASP* vasodilator-stimulated phosphoprotein, *WASP* Wiskott–Aldrich syndrome protein, *WAVE*  regulatory complex

Vasculogenesis is the development of a primary blood system during embryogenesis, via the de novo formation of blood vessels [1]. Vessels develop when endothelial precursor cells in the embryonic mesenchyme form an aggregate, which mature into small, single-layered endothelial tubes [2]. The primary blood vessels formed in this process are stable and allow blood flow in the developing embryo. However, these vessels have poor functionality and require further re-modelling via angiogenesis. Sprouting angiogenesis is guided by endothelial tip cells and promotes the expansion of existing vessels [2]. Tip cells are distinct from endothelial stalk cells by their position, dynamic filopodia and migratory behaviour, which defines the direction of new sprout growth [3]. The existing vessel wall is disassembled by enzymatic degradation, for example by matrix metalloproteinases, of the extracellular matrix (ECM) architecture and basal lamina. Endothelial tip cells migrate into the perivascular layer towards an initial chemotactic factor, such as vascular endothelial growth factor (VEGF) or Notch signalling, followed by endothelial cell invasion of the ECM [2, 3]. Endothelial stalk cells proliferate to facilitate vessel sprouting, enabled by the loss of cell-to-cell contact, and the lumen capillary begins to form by stalk cell coalescence [2]. Once the vessel has fully expanded, endothelial cell proliferation and migration are inhibited by anti-angiogenic factors. The basal lamina reconstitutes, the vessel wall re-assembles and the remodelled vessels then re-stabilise and mature by recruiting pericytes or vascular smooth muscle cells (VSMCs) [2]. Together, these cells encase the endothelial tube, protecting the ECM and supporting the vessel [2]. As such, the migration and proliferation of endothelial cells and VSMCs are critical processes in the development of functional vascular networks and are known to be influenced by the activity of small Rho GTPases (Fig. 1).

The dedicator of cytokinesis (DOCK) protein family has been implicated in critical cellular processes such as cell migration and adhesion, due to its role in the regulation of small Rho GTPases. Until recently, the DOCK family has been principally studied in relation to neuronal development, identifying a major role for DOCK proteins in neuronal cell functions and neurological disease [4–10]. Advances in our understanding of DOCK protein function in brain development and neurological disease has been previously published [11] so will not be further reviewed here. However, research over the last decade has determined that multiple DOCK proteins also function within the vascular system both experimentally and, critically, in the context of human disease. Taken together, 64 % (7/11) of DOCK proteins have now been demonstrated to regulate vascular processes, suggesting that further investigation of this protein family in pertinent vascular cell types is both important and timely. This review collates the existing evidence supporting a role for the DOCK family proteins in vascular development and cardiovascular disease, providing a specific perspective to establish a framework for directed research in this important field.

### Small Rho GTPases

Rho GTPases function as molecular switches, cycling between their active guanosine-5′-triphosphate (GTP)-bound and inactive guanosine diphosphate (GDP)-bound states. The best characterised of these are RHOA, ras-related C3 botulinum toxin substrate 1 (RAC1) and cell division cycle 42 (CDC42) [12]. Rho GTPase activity is controlled by the opposing actions of guanine nucleotide exchange factors (GEFs), GTPase-activating proteins (GAPs) and guanine nucleotide dissociation inhibitors (GDIs). GEF regulators activate GTPases by promoting the exchange of GDP for GTP. The Rho GEF family comprises approximately 80 members, divided into typical (Dbl) and atypical (DOCK) categories, and has been most commonly implicated in the development of neurological disorders and cancers [11, 13, 14]. Conversely, the GAP family, consisting of around 60 members, catalyse the intrinsic GTPase activity of small Rho GTPases, leading to their inactivation [15, 16]. In a similar fashion, GDIs bind to and stabilise GDP-bound small GTPases, sequestering them in the cytoplasm and therefore preventing the spontaneous GEF-catalysed guanine nucleotide exchange reaction [15].

GEF-mediated activation of Rho GTPases contributes to dynamic actin cytoskeletal assembly and rearrangement, forming the basis of cell-to-cell adhesion and migration [17, 18]. CDC42 is a small Rho GTPase present in most eukaryotes and is known for its role in the regulation of actin-based morphogenesis and cell polarity [19]. By directing filopodia formation, CDC42 modulates cell adhesion, migration and invasion in many cell types, including neurons, endothelial cells and VSMCs [17, 18]. CDC42-driven filopodia formation at the leading edge of endothelial tip cells has been shown to be required for angiogenesis and vessel patterning through the development of membrane protrusions, micropinocytosis, endothelial cell barriers, adherens junctions and tube formation [17–21]. In vascular development, filopodia emerge from endothelial tip cells in response to CDC42 activation, to regulate cell shape and migration, vascular branching and sensing of the microenvironment [22]. Filopodia are produced by the polymerisation of unbranched actin filaments, which are then arranged in parallel bundles at the filopodium tip [23]. This CDC42-induced localised actin polymerisation at the cell membrane promotes filament elongation via numerous pathways; CDC42 directly activates formin family proteins to nucleate and advance actin filament extension, whereas activation of Wiskott-Aldrich syndrome proteins (WASPs) up-regulates the actin-related protein 2/3 (ARP2/3) complex to produce branched actin filament networks [24]. Actin elongation is stimulated by the activation of uncapping proteins, for example, vasodilator-stimulated phosphoprotein (VASP), delivered to the growing filopodium tip by molecules such as myosin X [21]. CDC42 may also activate the IRSp53 adapter protein, which links actin to the membrane and promotes clustering of the uncapping protein (Fig. 1) [25, 26]. Filopodia formation is a dynamic process owing to the continuous assembly and disassembly of the actin filaments. Actin disassembly at the filopodium tip is typically cofilin- or gelsolin-mediated, but may also be driven by actin depolymerisation and retraction forces applied to the filopodium actin filaments by non-muscle myosin II. Interestingly, several molecules are common to both neural and vascular development, for example, neuropilin-1 (NRP1) is required for tip cell guidance in the developing central nervous system (CNS) as well as for CDC42-mediated filopodia formation in endothelial tips cells [27, 28]. To facilitate cell migration, filopodial protrusions must be stabilised by adherence of the filopodia to the ECM via focal adhesions (FAs). In both neuronal growth cones and endothelial tip cells, this mechanism physically links the actin cytoskeleton to the ECM via integrins to mediate cell advance.

In a similar fashion, RAC1 mediates lamellipodia formation, which is required for blood vessel development and has been shown to promote migration, adhesion, angiogenic sprouting and the permeability responses of endothelial cells to VEGF both in vivo and in vitro [17, 18, 29, 30]. Like CDC42, RAC1-mediated lamellipodia formation may be driven by the activation of the WASP-related WAVE regulatory complex, which promotes actin filament nucleation [31]. The ARP2/3 complex is stimulated to advance branched actin nucleation, whilst filament length is controlled by elongation factors, such as VASP and formin family members [31]. The serine/threonine-protein kinase (PAK) family contributes towards actin polymerisation in both filopodia and lamellipodia formation, and becomes active only when bound to either RAC1 or CDC42 [32]. PAKs have many downstream effectors, including LIM kinase (LIMK), to promote actin polymerisation and bundling [32]. As with filopodia, lamellipodial actin depolymerisation is propelled by severing proteins, such as cofilin (Fig. 1).

Further support for CDC42 involvement in mammalian vascular development is demonstrated by its conserved function in zebrafish, where Cdc42 is activated by pro-angiogenic Arfgef9b and has been found to regulate filopodia and endothelial cell guidance mechanisms during angiogenesis [33]. In mice, compromised blood vessel formation during development is seen in *Cdc42* knockout and endothelial-specific deletion models [18]. Moreover, in the mouse retinal vasculature, CDC42 activation has been associated with the pro-angiogenic factor NRP1, demonstrating inhibition of cellular protrusion upon *Nrp1* and *Cdc42* knockdown, a finding that has been replicated in zebrafish models [28]. Both CDC42 and RAC1 have also been shown to be essential for vascular development in vitro. Whilst CDC42 is required for protrusion formation and angiogenic sprouting, *RAC1* knockout endothelial cells are unable to contribute towards important angiogenic processes, such as tip cell migration, owing to their inability to form lamellipodia and FAs [29]. This is exemplified by *Rac1*-deficient mice, which are embryonic lethal by embryonic day 9.5 (E9.5), most likely due to defective development of major blood vessels and lack of small vessels observed in the embryo [29]. Therefore, through their respective functions in controlling actin cytoskeleton reorganisation, both CDC42 and RAC1 demonstrate roles in key cellular processes required for vasculogenesis and angiogenesis.

### The DOCK protein family

**Fig. 2:**
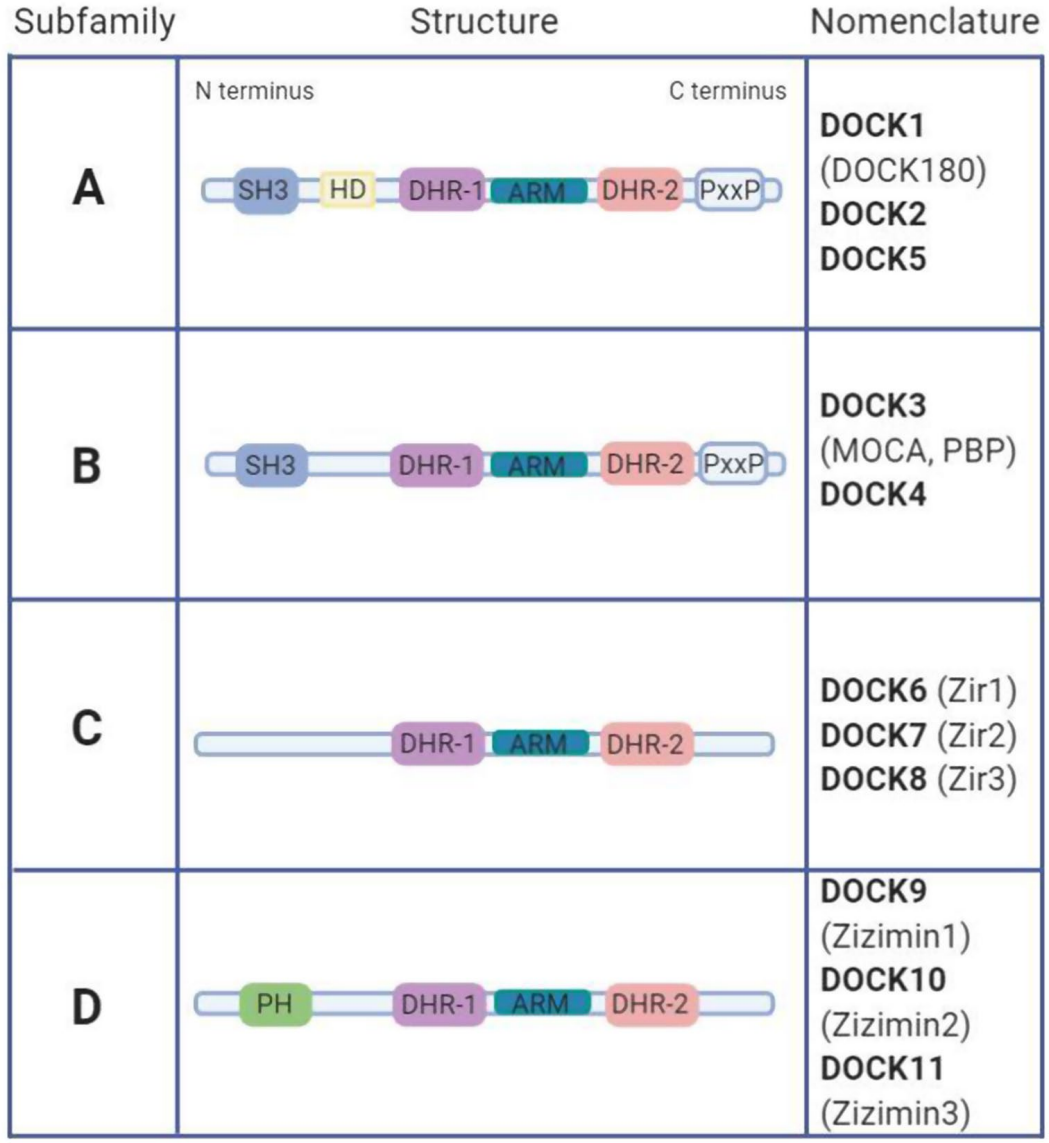
Structures of the DOCK protein subfamilies **a**–**d**. The DOCK protein family comprises 11 proteins, grouped into four subfamilies. Members of each subfamily share similar protein structure and functional domains. *ARM* armadillo repeat, *DHR* DOCK homology region, *DOCK* dedicator of cytokinesis, *HD* helix domain, *PxxP* proline-rich region, *PH* pleckstrin homology domain, *SH3* Src homology 3 domain

The DOCK proteins are a family of Rho GEFs known to regulate the small Rho GTPases CDC42 and RAC1. The family is subdivided into four groups, depending on their GTPase specificity and functional domains (Fig. 2). The DOCK-A (DOCK1, -2, and -5) and DOCK-B (DOCK3 and -4) subfamilies are generally specific for RAC1 activation and share the highest protein structure similarity of all DOCK subfamilies, including an N-terminal Src homology 3 (SH3) domain, two DOCK homology regions (DHR-1 and DHR-2), an armadillo repeat motif and a C-terminal proline-rich domain [34–38]. In addition, DOCK-A proteins, but not DOCK-B, contain a helical domain towards their N-terminus. The DOCK-C subgroup (DOCK6, -7 and -8) regulate the activity of both RAC1 and CDC42 [39, 40]. The only recognisable functional domains of this subset are the DHR-1 and -2 domains and armadillo repeat. Finally, the DOCK-D subfamily (DOCK9, -10 and -11) all contain an N-terminal pleckstrin homology (PH) domain, which binds phosphoinositides to facilitate DOCK-D protein translocation to the membrane. The DOCK-D proteins generally display CDC42 specificity, although both DOCK9 and -10 have also been implicated in RAC1 activation [35, 38, 41–43].

Unlike other Rho GEFs, DOCK proteins are not reliant upon PH and Dbl homology (DH) domains for GEF activity and membrane localisation [36, 39]. Rather, DOCK proteins are structurally related by their conserved functional DHR-1 and DHR-2 domains. DHR-1 mediates the localisation of the protein to the membrane via lipid, namely phosphatidylinositol (3,4,5)-trisphosphate (PIP_3_), interaction. PIP_3_ is a product of phosphatidylinositol 3-kinase (PI3K) activation, a key pathway in the regulation of proliferation, migration and adhesion, with a well-documented role in angiogenic processes through its phosphorylation of protein kinase B (AKT) [44]. At the plasma membrane, the DHR-2 domain binds the target small Rho GTPase to catalyse GTP-GDP exchange, leading to its activation and thus initiating the cascade of Rho GTPase effector proteins resulting in actin polymerisation and, in turn, lamellipodia and filopodia formation (Fig. 1) [36, 37]. The DHR-2 region comprises three lobes, known as A, B and C [45]. Through extensive contact with lobe B, lobe A stabilises the domain, whereas lobes B and C contain the GTPase binding site and catalytic centre to mediate guanine exchange of the target small Rho GTPase [37, 46]. Lobe B is comprised of two anti-parallel sheets and lobes A and C are near-identical anti-parallel alpha helical bundles, each consisting of four alpha-helices [45]. The SH3 domain, shared by the DOCK-A and -B subfamilies, binds to the PH domain of engulfment and cell motility (ELMO) proteins, required for activation of DOCK GEF activity [47–50]. The DOCK-A and -B C-terminal proline-rich region has been found to bind phosphatidic acid (PA) as well as SH3-containing adaptor proteins, such as CRK, which contribute towards the functional complex required for DOCK-mediated Rho GTPase activity [48, 51].

To date, DOCK3, -8, -10 and -11 have not been associated with vascular development and remain primarily implicated in the pathogenesis of neurological disease and/or immune-related disorders (Table 1). By contrast, mounting evidence supports an argument for the importance of the other DOCK family members in critical vascular processes, potentially implicating the wider DOCK protein family in the development of vascular disease.

**Table 1:** Summary of key research and human diseases associated with the DOCK protein family

DOCK protein and subfamily	Rho GTPase specificity	Human tissue expression	Cardiac/vascular involvement	Mouse model	Zebrafish model	Associated diseases	Other system involvement
DOCK1 (A)	Rac1	All tissues except peripheral blood leukocytes (localised to nucleoplasm)	Role for DOCK1 in vitro in endothelial cell migration and membrane ruffling in response to CXCL12 [51]	Lethal before weaning; Severe edema, CV defects, submembranous VSD and DORV [51, 56]	Altered trunk morphology, strong vascular defects; Defective myoblast fusion; Impaired myelination of peripheral axons [55, 63, 102, 103]	Down-regulated in intraosseous vascular malformation (VMOS), caused by biallelic *ELMO2* mutation; Dysregulated in various cancers [60, 104]	Nervous system
DOCK2 (A)	Rac1	Peripheral blood leukocytes, thymus, spleen, small intestine and colon (cytoplasmic localisation)	Promotes VSMC migration and proliferation in vitro; High *DOCK2* expression in neointima formation after vein graft [74, 75]	Reduced amyloid burden in Alzheimer’s mouse model; Defective T and B lymphocyte migration [34, 105]	ND	Alzheimer’s disease; Early-onset invasive infections; Over-expressed in chronic lymphocytic leukaemia [11, 76, 104, 105]	Immune system; Nervous system
DOCK3 (B)	Rac1	Brain, CNS (cytoplasmic localisation)	ND	Axonal degeneration, motor deficiencies with abnormal ataxic gait and impaired learning [106, 107]	ND	Alzheimer’s disease; Attention deficit hyperactivity disorder; Developmental delay and hypotonia [11, 107]	Nervous system
DOCK4 (B)	Rac1/Rap1	Widespread expression, highly expressed in neutrophils (localised to nucleoli, plasma membrane, cytosol and Golgi apparatus)	Controls lumen formation and tubule remodelling; *mir-21* (associated with cardiac hypertrophy and fibrosis) causes *DOCK4* down-regulation in VSMCs in vitro, mediating loss of VSMC contractility [79]	Early embryonic lethality, decreased blood vessel lumen diameter [80]	ND	Autism; Dyslexia; Myelodysplastic syndrome; Prostate and ovarian cancer; Schizophrenia [11, 13, 81]	Nervous system
DOCK5 (A)	Rac1	Widespread expression, highly expressed in neutrophils (cytoplasmic localisation)	*mir-21* causes *DOCK5* down-regulation in VSMCs in vitro and mediates loss of VSMC contractility [79]	High bone mass; Cataract development [84, 108]	Defective fast myoblast fusion [102]	Parkinson’s disease; Acute myeloid leukaemia; Head and neck squamous cell carcinoma [11, 104]	Myoblast fusion; Intestinal epithelial cell spreading and migration
DOCK6 (C)	Rac1/Cdc42	Widespread expression (granular cytoplasmic localisation)	Role in migration of VSMCs in vitro; Development of cardiac and vascular defects in DOCK6-mediated AOS [88, 93]	Defective axon extension both in developmental stages and after injury [7]	ND	Adams-Oliver syndrome (AOS) type 2 [86]	Nervous system
DOCK7 (C)	Rac1/Cdc42	Widespread expression (localisation unknown)	*mir-21* causes *DOCK7* down-regulation in VSMCs in vitro [79]	Impaired neuroblast migration; Affects myelination by Schwann cells, increases myelin thickness in sciatic nerves [109, 110]	ND	Early infantile epileptic encephalopathy (EIEE23) [6]	Nervous system
DOCK8 (C)	Rac1/Cdc42	Widespread expression, enhanced in blood and lymphoid tissue (cytoplasmic/nuclear localisation)	ND	Defective dendritic cell migration; Hyper-responsive immunity in asthma models [111, 112]	ND	Autism; Autosomal recessive hyper IgE syndrome (DOCK8 deficiency); Mental retardation [11, 13]	Nervous system; Immune system
DOCK9 (D)	Rac1/Cdc42	Widespread expression, enriched in immune cells (cytoplasmic localisation)	Role in lateral and tip filopodia formation and in vessel branching [80]	No apparent phenotype [96]	ND	Bipolar disorder; Keratoconus, corneal disorder [11, 13]	Nervous system
DOCK10 (D)	Rac1/Cdc42	Widespread expression, enhanced in brain and lymphoid tissue (cytoplasmic localisation)	ND	Reduction in B cell count, impaired macrophage migration [96]	ND	Autism [11]	Nervous system; Immune system
DOCK11 (D)	Cdc42	Widespread expression (nuclear localisation)	ND	Impaired macrophage migration [96]	ND	ND	Immune system

*CNS* central nervous system, *CV* cardiovascular, *DORV* double outlet right ventricle, *KD* knockdown, *KO* knockout, *ND* no data, *VSD* ventricular septal defect, *VSMC* vascular smooth muscle cell

### DOCK1

**Fig. 3:**
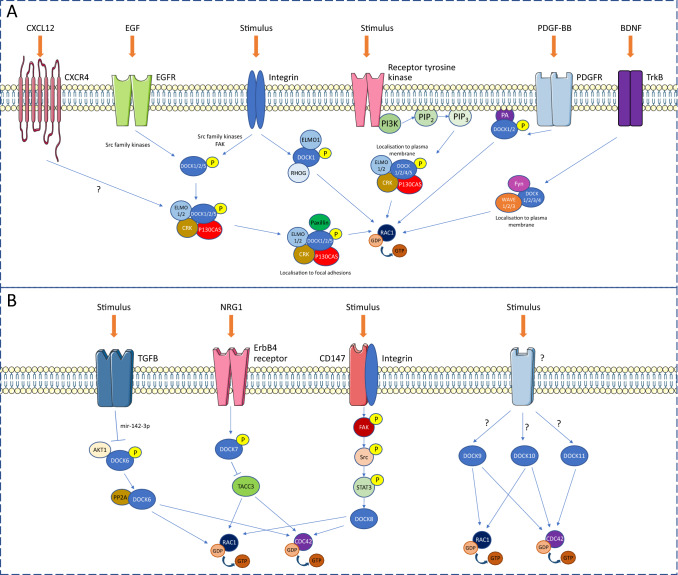
DOCK protein family-specific signalling pathways regulating CDC42 and RAC1 activity. **a** Overview of the receptors, signalling pathways and binding complexes regulating the DOCK-A and -B subfamilies. Activation of DOCK proteins 1–5 results in the guanine nucleotide exchange of the small Rho GTPase RAC1, leading to its activation. **b** Known regulatory pathways of proteins in subfamilies DOCK-C and -D. Stimulation of DOCK proteins 6–11 initiates activation of RAC1, CDC42, or both. *BDNF* brain-derived neurotrophic factor, *CXCL12* C-X-C motif chemokine 12, *CXCR4* C-X-C motif chemokine receptor type 4, *DOCK* dedicator of cytokinesis protein, *ELMO1* engulfment and cell motility protein 1, *EGF* epidermal growth factor, *EGFR* epidermal growth factor receptor, *FAK* focal adhesion kinase, *GDP* guanosine diphosphate, *GTP* guanosine triphosphate, *NRG1* neuregulin 1, *P* phosphate, *PA* phosphatidic acid, *PDGF-BB* platelet-derived growth factor subunit B, *PDGFR* platelet-derived growth factor receptor, *PI3K* phosphatidylinositol 3-kinase, *PIP2* phosphatidylinositol (4, 5)-biphosphate, *PIP3* phosphatidylinositol (3, 4, 5)-triphosphate, *PP2A* protein phosphatase 2, *STAT3* signal transducer and activator of transcription 3, *TACC3* transforming acidic coiled-coil-containing protein 3, *TGFB* transforming growth factor-beta, *TrkB* tropomyosin receptor kinase B, *WASP* Wiskott–Aldrich syndrome protein, *WAVE* regulatory complex

DOCK1, formerly named DOCK180 due to its 180 kDa protein mass, was the first of the DOCK family proteins to be characterised [52]. Mammalian DOCK1 protein expression is distributed throughout all tissues, and enriched DOCK1 expression has been identified in pulmonary artery endothelial cells, human umbilical vein endothelial cells (HUVECs) and human microvascular endothelial cells (HMVECs) [16, 53, 54]. Despite exhibiting relatively low tissue specificity, DOCK1 plays a specific role in the formation of skeletal muscle, as demonstrated in zebrafish, fruit fly and mouse models [55, 56]. This activity is regulated on a cellular level by the formation of several regulatory complexes between DOCK1 and different binding partners (Fig. 3a). Of particular interest, DOCK1, as well as DOCK2, -4 and -5, interacts with ELMO1 or -2, whereby the binding of ELMO1/2 induces a conformational change in DOCK1, releasing an auto-inhibitory mechanism and enabling DOCK1 to act as a RAC1-specific GEF [35, 36, 55]. Once associated with DOCK1, ELMO1 can interact with RHOG, promoting translocation of DOCK1 to the plasma membrane, where the DOCK1/ELMO1/RHOG complex activates RAC1 [9, 57]. Alternatively, DOCK1 translocation from the cytosol to the cell membrane can be mediated by interactions between the DOCK1 DHR-1 domain and membranous PIP_3_ [36, 58]. Upon membrane localisation, DOCK1 activates RAC1 to facilitate RAC1-dependent actin reorganisation at the leading edge of the cell, which is required for directional cell movement [36, 59].

Conversely, ELMO2 has been found to recruit and complex with DOCK1 at initial cell-cell contacts in Madin-Darby canine kidney cells [50]. The DOCK1/ELMO2 complex is essential for rapid recruitment and spreading of E-cadherin, actin reorganisation, localised RAC1 activity and strong cell-cell adhesion. In a vascular context, loss-of-function mutations in *ELMO2* can cause autosomal recessive intraosseous vascular malformation (VMOS), which results in malformed blood vessels lacking a mature vascular smooth muscle layer [60]. Analysis of VMOS fibroblasts identified a correlation of ELMO2 deficiency with the down-regulation of DOCK1, resulting in impaired RAC1-dependent cell migration [60].

Further involvement of DOCK1 in cell migration may be partially regulated in a VEGF-independent manner, as telomerase-immortalised HMVECs showed increased motility following polypyrimidine tract binding protein 1 (PTPB1) inhibition, through activation of the DOCK1/RAC1 pathway [61]. PTPB1 is known to impede VEGF receptor (VEGFR) signalling in endothelial cells, resulting in decreased cell motility [62]. Upon integrin stimulation at the membrane, FAK, epidermal growth factor receptor (EGFR) and platelet-derived growth factor receptor (PDGFR) induce DOCK1 phosphorylation by Src family kinases, promoting the association of DOCK1 with the P130CAS (BCAR1) and CRK scaffold proteins (Fig. 3a) [54]. Interestingly, FAK, P130CAS and DOCK1 have all been implicated in axonal outgrowth, driven by DCC receptor interaction in netrin-1 (NTN1) signalling, an established angiogenic pathway [5, 63]. Under PTPB1 inhibition, DOCK1-P130CAS-CRK complex formation is promoted, leading to increased RAC1 activity. Alternatively, the DOCK1-P130CAS-CRK complex can be recruited to FAs by further association with FA-essential paxillin, wherein RAC1 activation specifically drives increased migration of human glioma cells [54]. In human microvascular cells, this paxillin-CRK-DOCK1 complex was found to be disassembled by TIMP metallopeptidase inhibitor 2 (TIMP2) in favour of paxillin-CRK-C3G complex formation, switching GTPase activation from RAC1 to RAP1 [64]. As TIMP2 is a suppressor of migration and impedes DOCK1-mediated cell motility, these data imply a potentially global role for DOCK1 in cell migration, further substantiated by siRNA-mediated *DOCK1* knockdown studies in HUVECs and intestinal epithelial cells [65]. In this study, *DOCK1* depletion reduced cell spreading, cell migration and lamellipodial extensions at the migratory edge of the cell, indicating that reduced RAC1 activation caused by *DOCK1* knockdown is the primary cause for these phenotypes. These findings were mimicked by *DOCK5* depletion in the same study [65]. The high amino acid conservation within DHR-1 domains across the DOCK family may point towards a common PIP_3_-associated mechanism for mediating cell migration in DOCK-expressing cell types. Interestingly, the DOCK1/ELMO1 complex has also been found to associate with the Dbl-RhoGEFs Intersectin-1 and -2 (ITSN1/2), known to contribute to intercellular endothelial adhesions [66]. DOCK1 is the only DOCK protein known so far to interact with typical RhoGEFs and, when complexed, DOCK1/ELMO1-ITSN1/2 directly interacts with G-protein coupled receptor 124 (GPR124) to aid cell adhesion [66]. As GPR124 is associated with brain angiogenesis and blood-brain-barrier integrity, and has been shown to regulate CDC42-mediated filopodia formation in human brain vascular pericytes [67], these observations provide further evidence to implicate both typical and atypical Rho GTPase regulators in vascular development.

A relationship between DOCK1 and the cardiovascular system in vivo was first observed in 2010, when *Dock1*-deficient mice were observed to have developed large edema at E14.5 [51]. Upon dissection of *Dock1*-deficient embryos, defects in cardiovascular development were identified, namely sub-membranous ventricular septal defect and double outlet right ventricle [51]. These cardiac abnormalities were noted to resemble the phenotype observed with knockdown of chemokine receptor type 4 (*Cxcr4*), a gene encoding the receptor for CXCL12 and known to be critical for vascular development and endothelial cell morphology and branching [68–70]. Co-expression of *DOCK1* and *CXCR4* in cardiac and vascular endothelial cell lineages suggested a possible role for DOCK1 in endothelial cellular processes, such as cell invasion of the ECM, although it is currently unclear whether DOCK proteins are activated during endothelial ECM cell invasion [51]. Of interest, CDC42 is a key player in the formation of invasive podosomes, through interaction with its downstream effectors WASP and ARP2/3. However, the role of DOCK proteins in this process remains to be delineated (Fig. 1) [71]. Subsequently, investigation of DOCK1 function in vascular endothelial cells using chemotaxis assays identified a role for DOCK1 downstream of CXCR4 in cardiac endothelial cell migration and membrane ruffling in response to CXCL12. As CXCL12-induced activation of CXCR4 leads to association with ELMO1 to promote recruitment and activation of DOCK1 to the plasma membrane [72], these findings point towards a mechanism of action whereby CXCR4 stimulates DOCK1-mediated RAC1 activation to control endothelial cell migration and invasion via actin cytoskeleton reorganisation during cardiovascular development [51].

Consistent with the vascular phenotype in *Dock1*^−/−^ mice, morpholino-directed *dock1* knockdown in zebrafish induced notable vascular defects, but only at higher oligo concentrations [55]. It is unclear whether this observation was a secondary effect derived from the known Dock1 function in myoblast fusion, leading to trunk defects upon *dock1* knockdown [55]. Nonetheless, the shift in zebrafish *dock1* expression from a ubiquitous pattern during early somitogenesis, to a more specific vascular expression in the dorsal aorta and posterior cardinal vein later in development, would support a potential role during vascular development [55]. This temporal and spatial expression pattern is mimicked by *elmo1*, which demonstrates non-specific expression throughout the embryo during early somitogenesis and adopts a vasculature- and CNS-specific pattern by 24 hours post fertilisation [55]. Investigation of *elmo1* activity in early zebrafish development confirmed its important role in the developing vasculature, with morpholino knockdown inducing multiple vascular-related phenotypes, including heart edema, accumulation of blood in the yolk sac, deformation of the intersomitic vessels and agenesis of the dorsal longitudinal anastomotic vessel [55]. Of note, this study identified Ntn1 as an upstream activator of the Elmo1/Dock1/Rac1 signalling cascade in zebrafish, confirming the Ntn1/Dock1 interaction in a vascular context as well as in axonal outgrowth, as discussed above [5, 55]. Additional zebrafish studies have demonstrated the effects of Dock1 and Elmo1 overexpression in reducing endothelial cell apoptosis and inducing angiogenesis, further supported by HUVEC studies confirming the same results under DOCK1 and ELMO1 overexpression and identifying activation of the pro-survival PI3K/AKT signalling cascade by DOCK1 and ELMO1 [63]. As DOCK1-mediated GEF activity is contingent on formation of an ELMO1/DOCK1 complex in both zebrafish and mammalian systems, these observations indicate an essential mechanism of ELMO1/DOCK1-mediated RAC1 activation during vascular development.

Although not yet implicated in human vascular disease, these combined data imply a conserved role for DOCK1 in the control of mammalian cardiac development and vascularisation, under the regulation of multiple, vascular cell-specific genes.

### DOCK2

In stark contrast to DOCK1, which is expressed throughout all tissue types aside from peripheral blood leukocytes, DOCK2 expression is confined predominantly to haematopoietic cells, but is confirmed to also have low-level expression in HMVECs [53, 59]. DOCK2 shares 63.2% identity with DOCK1, excluding the carboxyl terminal variable regions [45]. Unlike DOCK1, induction of DOCK2 expression in normal rat kidney cells led to a rounding and detachment phenotype in vitro, thought to be caused by RAC1 activation [59]. Despite both proteins driving RAC1 activation, it is thought that the different morphological effects observed with DOCK1 and DOCK2 over-expression may stem from alternative intracellular localisation of these proteins [59]. However, this is yet to be fully elucidated, and different, cell-specific mechanisms of DOCK1 and -2 activation may also contribute to the disparate cellular responses.

Known for its involvement in immune regulation, DOCK2 regulates lymphocyte activation and migration [34, 73]. This has been demonstrated in a *Dock2*^−/−^ mouse model, where T and B lymphocyte migration was inhibited, and RAC1 activation and actin polymerisation were virtually abolished [34]. Similar to DOCK1, DOCK2 is regulated by ELMO1 which drives binding of DOCK2 to PIP_3_. PIP_3_ association translocates DOCK2 to the membrane in a PI3K-dependent manner, leading to RAC1 activation which in turn serves as a positive feedback loop, stabilising PIP_3_ at the cell’s leading edge.

Interestingly, DOCK2 has also been implicated in vascular remodelling [74], wherein VSMCs de-differentiate from a contractile to a synthetic phenotype, triggered by damage to the blood vessel walls and followed by VSMC migration and proliferation. This can lead to the narrowing of the blood vessel lumen. Low endogenous DOCK2 expression is maintained in VSMCs, but is drastically increased by the addition of platelet-derived growth factor subunit B (PDGF-BB), a molecule known to stimulate VSMC phenotypic modulation [74]. Stimulation of DOCK2 expression correlates with a decrease in the expression of VSMC marker proteins, such as serum response factor (SRF) and Mycod. Conversely, *DOCK2* knockout was shown to increase VSMC marker protein expression, supporting an argument for the requirement of low level *DOCK2* expression in VSMCs to maintain contractile protein homeostasis [74]. A further study has implicated a role for DOCK2 in neointima formation, which is the generation of scar tissue in the blood vessel lumen after vein graft [75]. This was identified when unusually high levels of *DOCK2* expression were detected in vein grafts of male Sprague-Dawley rats that underwent jugular vein-carotid artery bypass grafting [75]. Adding to existing knowledge of the role of DOCK2 in VSMC differentiation, *DOCK2* knockdown was noted to inhibit rat VSMC migration and proliferation, whereas over-expression significantly increased these processes [75]. As VSMC migration and proliferation are key processes in angiogenesis, these findings point towards a role for DOCK2 in the maintenance of healthy mammalian blood vessels. To further validate the involvement of DOCK2 in vessel maintenance, the knockdown of endogenous *DOCK2* in grafted veins was shown to reduce neointimal formation and lead to improved hemodynamics in vein grafts [75]. Despite this clear association with vessel maintenance, DOCK2 has not yet been implicated in human vascular disease, but has been reported to cause an inherited immunodeficiency disorder [76].

### DOCK4, -5 and -7

DOCK4 demonstrates diverse tissue expression, including in HUVECs and HMVECs, and has been observed to regulate the activity of both RAC1 and, in contrast to other DOCK family members, RAP1 [16, 53, 77]. RAC1 activation requires the DHR-2 domain and DOCK4 binding to the PH domain-containing protein ELMO1/2, similar to other DOCK-A and -B subfamily members [78]. However, the DOCK4 SH3 domain has a unique role in negatively regulating RAC1 activity [78]. The DOCK4 functional domains are also important in protein expression localisation, as exemplified in a splice isoform of *DOCK4* lacking exon 49, which contains a conserved region [77]. Tissue expression of the alternatively spliced isoform was restricted to the stereocilia of the cochlea and retina, as opposed to the usual widespread expression of full-length DOCK4 in a diverse range of tissues [77].

Analogous to other DOCK family proteins, DOCK4 is known to contribute towards cell migration [8, 78–80]. This has been demonstrated in fibroblast cells, where DOCK4 activated RAC1 at the cell membrane [78]. In this study, over-expression of wild-type *DOCK4* was found to significantly increase cell migration, by comparison to constructs containing either a C-terminal or DHR-2 domain mutation.

Several reports have implicated DOCK4 involvement in vascular-type cells, noted initially when the microRNA *mir-21* exhibited downstream effects on *DOCK4*, *DOCK5* and *DOCK7* expression in VSMCs [79]. *mir-21* is known to play a critical function in the development of cardiac hypertrophy and fibrosis, and influences cell proliferation and migration through targeting the knockdown of downstream effectors [68]. Speculation for the role of *mir-21* in vascular development is strengthened by its association with bone morphogenetic protein (BMP) and transforming growth factor-beta (TGF-β) signalling, known to promote a contractible phenotype in VSMCs by targeting the programmed cell death 4 (*PDCD4*) gene [79]. Indeed, upon *mir-21* over-expression in pulmonary artery smooth muscle cells, DOCK proteins 4–7, 9 and 10 were found to be down-regulated, indicating a notable association between the DOCK family of proteins and this cell type [79]. Further investigation using siRNA identified a significant decrease of VSMC migration upon knockdown of *DOCK4*, -*5* and -*7*, and a reduction of VSMC contraction upon *DOCK4* and *DOCK5* knockdown, but not *DOCK7*. This suggests a specific function for DOCK4 and -5 in the regulation of vascular cell contractility, substantiated by the elevated expression of contractile genes upon siRNA targeting of DOCK4 and -5 [79]. As these proteins contain multiple SH3 domains which are not present in DOCK7, it is possible that cytoskeletal remodelling may be mediated through SH3 domain interactions with other signalling molecules.

DOCK4 involvement in vascular development was further consolidated in a more physiologically relevant study, using siRNA-based screens in an organotypic angiogenesis system which recapitulates endothelial cell association, vessel sprouting and tubule establishment [80]. Here, the role of DOCK4 was investigated in 3D culture of endothelial cells seeded onto a confluent layer of fibroblasts. Knockdown with *DOCK4*-specific siRNA led to a marked decrease in the number of vessel branches that developed, suggesting that DOCK4 promotes vessel sprouting. The remaining developing tubules were thinner and with fewer lateral cell-to-cell contacts, implicating DOCK4 in tubule development and endothelial cell adhesion, a critical process in angiogenesis [80]. To further underline the importance of DOCK4 in cell adhesion, siRNA-mediated *Dock4* depletion in normal mouse osteoblasts elicited a marked suppression of adherens junctions [81]. When examined in vivo, homozygous *Dock4* knockout in the mouse caused early embryonic lethality, however heterozygous *Dock4*^+/−^ mice exhibited a variety of angiogenic-associated defects, including a decrease in the blood vessel lumen size, seen in the brain parenchyma of E13.5 embryos [80]. This decrease in lumen size was not associated with pericyte coverage of the vessel, suggesting that another mechanism may be affected, for example cell contractility or motility [79, 80]. Combined, these results provide evidence indicating a role for DOCK4 in endothelial cell protrusive activity, tubule remodelling, organisation of lateral contacts and lumen formation.

In the context of vascular disease, DOCK4 was recently implicated in a mechanism for atherogenesis, the formation of arterial fatty deposits, and in neovascularisation following an ischaemic event [82, 83]. Specifically, DOCK4 was noted as a critical interacting partner of scavenger receptor class B type 1 (SR-B1), a receptor observed in mice to bind LDL and mediate its delivery into the arteries, instigating the cascade towards the development of atherosclerosis [82]. Co-expression of SR-B1 and DOCK4 in vivo was observed in the aortic endothelium of mouse models, and increased levels of both proteins detected in the aorta prior to atherosclerotic lesion formation [82]. *DOCK4* knockdown decreased the internalisation of SR-B1 in endothelial cells, impeding the delivery of circulating LDL into the sub-endothelial space by SR-B1 and therefore reducing atherosclerotic lesion formation [82].

Additional support for the potential role of DOCK5 in vascular development lies in the shared regulatory mechanisms acting on both DOCK1 and DOCK5. Like DOCK1, DOCK5 localises to FAs via a DOCK5-P130CAS-CRK complex, implying a similar role for DOCK5 in cell adhesion. Of interest, *Dock5* knockout mice display increased trabecular bone mass due to defective osteoclasts impeding microtubule-dependent podosome assembly [84]. However, DOCK5 interactions with ARF GTPase-activating protein 2 (GIT2) result in reduced GEF activities, in contrast to the positive regulation of DOCK1 by GIT1 and -2 for recruitment to FAs [85]. Although compelling, both DOCK5 and DOCK7 have yet to be thoroughly investigated in a vascular context, hence additional work is required to fully elucidate their respective regulatory mechanisms.

### DOCK6

DOCK6, alternatively named ZIR1, has been strongly associated with a role in neuronal cell migration [7, 39]. Nonetheless, *Dock6* demonstrates localised expression to the developing heart and growing limb buds in the E10.5- and E11.5-stage mouse embryo and is shown to be highly expressed in both HUVECs and HMVECs [16, 53, 86]. In a neuronal context, DOCK6 activity is known to be regulated by its phosphorylation state; DOCK6 GEF activity is inhibited by AKT-mediated phosphorylation at Ser1194, whereas Ser1194 dephosphorylation by phosphatase PP2A interaction promotes DOCK6-mediated RAC1 and CDC42 activation [7]. However, regulation of DOCK6-mediated GEF activity in a vascular context remains to be explored.

*DOCK6* was identified as the second causal gene of Adams-Oliver syndrome (AOS), a rare developmental disorder characterised primarily by the congenital absence of skin on the scalp, known as aplasia cutis congenita (ACC), and terminal transverse limb defects (TTLD), both of which present with a wide range of severity. These primary features may be associated with a wide array of additional clinical features, including cardiovascular, neurological and ocular defects. Importantly, congenital heart defects and cutis marmorata telangiectatica congenita (CMTC), a rare cutaneous vascular anomaly, are each observed in ~ 20 % of AOS cases [87]. *DOCK6* mutations underlie an autosomal recessive form of AOS and the vast majority are predicted to lead to a disruption of DOCK6 function, due to either premature protein truncation or degradation of mutant transcripts by nonsense-mediated mRNA decay. Consistent with a loss of GEF activity, patient-derived fibroblasts revealed actin cytoskeletal defects resembling CDC42 and/or RAC1 inactivation [86]. To date, ~ 20 independent *DOCK6* mutations have been described, accounting for 7 % of all reported AOS cases [88]. Predicted loss-of-function variants cover all mutation types including missense and nonsense, and are located across the length of the gene [10, 86, 89–91].

Patients with *DOCK6* mutations display a broad range of clinical presentations in addition to ACC and TTLD. Of significance, a large proportion of patients exhibit anomalies associated with the heart and/or vasculature, implicating AOS-related DOCK6 dysregulation in aberrant vascular development [88]. Loss-of-function *DOCK6* mutations are also strongly associated with CNS and structural eye abnormalities [10]. Furthermore, developmental delay and seizures have been identified in > 45 % of patients with microcephaly and, even in the absence of microcephaly, demonstrate strong correlation with the presence of impaired intracranial vascularisation [88]. Notably, abnormal pericyte recruitment, perhaps due to impaired pericyte migration, has been identified in post-mortem examination of AOS patients [92]. Ectasia and tortuosity of large veins, irregular medial thickness due to uneven mural cell deposition, and abnormal retinal vascularisation have all been observed [92]. Whilst the molecular causes of AOS in these patients remains unresolved, these characteristics may explain the observed clinical features in *DOCK6*-related AOS and suggest a role for the causal gene in the recruitment and migration of pericytes during angiogenesis [10, 86, 89, 90]. However, the functional impact of AOS-specific mutations in vascular cell types remains to be examined, thus providing much scope for future investigations.

Interestingly, DOCK6 has been implicated in the TGF-β signalling pathway, which is critical for many cellular processes including vascular development and homeostasis [93]. Importantly, TGF-β and BMP have roles in promoting VSMC differentiation and maintaining the contractile phenotype of VSMCs [94]. TGF-β signalling induces the haematopoietic cell-specific microRNA *mir-142-3p*, which has been shown to down-regulate *DOCK6* expression and simultaneously decrease cell migration in VSMCs, providing preliminary evidence for a role for DOCK6 dysregulation in disrupted vascularisation [93]. Transient *DOCK6* knockdown has been reported to exhibit a much more severe phenotype in HeLa cells by comparison to AOS patient-derived cells harbouring biallelic *DOCK6* mutations [86, 95]. This phenomenon may be partly explained by overlapping functions between DOCK family members, leading to genetic compensation upon prolonged gene loss and reducing the severity of the phenotype. While one study identified an increase in RHOA activity in *DOCK6*-negative patient-derived cells, potentially highlighting a compensation response mechanism, alterations in *DOCK* family gene expression upon stable *DOCK6* knockdown remain unexplored [95]. Therefore, further investigation into the functional effects of solely *DOCK6* knockdown on VSMCs is required. Nonetheless, the existing findings support an important role for DOCK6-mediated Rho GTPase dysregulation in the development of AOS-related cardiovascular and neurological defects.

### DOCK9

Similar to DOCK6, the DOCK-D subfamily member DOCK9 (also known as ZIZIMIN1) has been demonstrated to act as a GEF to both RAC1 and CDC42 [42, 43, 96]. In addition to strong associations with the nervous system, DOCK9 is implicated in vascular development and is expressed in HUVECs [16]. In human epithelial cells, DOCK9 has been documented to interact with SMAD2 and -3 in response to TGF-β1 stimulation, indicating a potential role as a downstream effector in the TGF-β-mediated regulation of cell motility [97]. VEGF promotes DOCK9 association with DOCK4 to influence cellular actin dynamics [80]. Through knockdown studies in 3D endothelial cell culture, DOCK9 has been shown to contribute towards the development of lateral and, to a lesser extent, tip filopodia [80]. More specifically, *DOCK9* depletion resulted in the formation of tubules with a more linear morphology than their wild-type counterparts. In an attempt to further delineate the role of DOCK9 in cell morphology, inducible expression of *DOCK9* was assessed in HeLa cells [43]. This resulted in reduced cell elongation and stress fibres, while inducing the formation of filopodia and membrane ruffles [43]. Taken together, these data indicate a requirement for DOCK9 in the remodelling of lateral organisation and the actin cytoskeleton in various cell types, including endothelial cells, and in vessel lumen morphogenesis [42, 43, 80].

## Discussion and conclusions

Existing research highlights migration and proliferation as key cellular processes influenced by the DOCK family of proteins, through its regulation of CDC42 and RAC1 activity. While most of these findings have been documented in a neuronal context, it is becoming increasingly clear that DOCK proteins play an essential part in the development and maintenance of the vasculature and other systems (Table 1). By comparison to the nervous system, our understanding of the pathways controlling DOCK protein activity within a vascular context remains limited. However, some mechanisms of action may be inferred from their roles in neurogenesis, owing to the parallelisms between neural and vascular development. Both neuritogenesis and angiogenesis are reliant upon single cell branching, and the productive advance of neural growth cones or vascular tip cells depends on integrin-mediated adhesions to the local ECM for guidance in sprout pathfinding. As discussed, angiogenic sprouting relies heavily on actin cytoskeleton reorganisation due to the actions of active CDC42 and RAC1 and their downstream effector proteins. In neuronal branching morphogenesis, CDC42 is activated in response to guidance molecules such as NTN1 and NRP1, which have each been shown to be required for tip cell guidance by CDC42 activation and filopodia formation both in the developing CNS and vasculature [5, 27, 28]. Hypothetically, given the substantive evidence associating the DOCK protein family with neurogenesis, the numerous commonalities between neuronal and vascular advances during development may be extrapolated to build a speculative bridge linking DOCK proteins enriched in human endothelial cells to a potential angiogenic role [16, 53].

A number of vessel-specific and pro-angiogenic interactors, such as CXCR4, NTN1 and BMP4, among others, have been associated with DOCK-mediated GEF activity in vascular cell types [51, 68, 79]. Of interest, members of the DOCK family are known to interact with proteins implicated in Notch signalling, for example DOCK3-Presenilin and DOCK7-TSC1/2 [98, 99]. Whilst the involvement of Notch signalling during vascular development is well-known, these DOCK-Notch pathway interactions have not yet been fully investigated in a vascular context. The role of the CXCR4/CXCL12 signalling pathway in DOCK protein regulation is of particular interest, owing to one of several known mechanisms of activation through the Delta-like canonical Notch ligand DLL4, demonstrating a potential cross-over between the DOCK and pro-angiogenic Notch signalling pathways, and its parallel involvement in the activation of certain Dbl-family Rho GEFs [68, 69, 100]. For example, the Dbl family member PIP_3_-dependent RAC exchanger 1 (PREX1) mediates angiogenic responses downstream of CXCL12 activity promoted by VEGF [101]. In this study, endogenous expression of PREX1 in endothelial cells was required for RAC1 activation, cell migration and in vitro angiogenesis in response to CXCL12 stimulation [101]. Considering the overlapping functions of the DOCK and Dbl GEF families in regulating the small Rho GTPases, it is tempting to speculate that these proteins may interact with CXCR4 in a similar manner and further implicate the pro-angiogenic VEGF pathway in the regulation of DOCK proteins.

The links between DOCK proteins and molecules associated with vascular development strongly imply functional importance of this family during angiogenesis. This postulation would be further supported by evidence of anti-angiogenic factors inhibiting DOCK protein activity. However, DOCK regulation by anti-angiogenic factors has not yet been thoroughly investigated. Despite the functions of DOCK proteins in actin cytoskeleton remodelling by regulation of RAC1 and CDC42, and the growing evidence implicating DOCK proteins in pro-angiogenic processes, current research indicates a defined role for VEGF receptors upstream of DOCK4 and -9 activity only [80]. Importantly, although various angiogenic factors have been noted to contribute towards DOCK protein activation, very little is known about how these molecules interact to facilitate DOCK GEF activity. Findings such as these suggest that DOCK protein modulation in the vasculature is currently under-researched and a stronger focus to elucidate the pathways influencing DOCK activity in vascular biology would be advantageous.

DOCK family members share a high degree of conservation, particularly across the DHR-1 and DHR-2 functional domains, and demonstrate overlapping tissue expression. Yet, pathogenic variation in *DOCK* family genes are associated with a diverse range of human disorders, including developmental, neurological and vascular abnormalities (Table 1). This review highlights that seven DOCK proteins (1, 2, 4–7 and 9) have key functions within the cardiovascular and/or neurological systems. The differences in clinical phenotypes may be due to temporal and spatial expression of numerous factors, including miRNAs, which restrict *DOCK* gene expression to either vascular- or neuronal-specific tissue [79, 93], or binding partners and DOCK protein regulators, leading to tissue-specific GEF activity [51, 55]. Conversely, alteration of conserved sites is known to change the expression pattern of some DOCK proteins [77]. It is possible that the less well-conserved functional domains, such as the SH3 and PH domains, could affect the localisation and binding activity of individual DOCK proteins and therefore lead to the range of phenotypes exhibited upon protein loss. However, this is yet to be explored.

The possibility that compensatory mechanisms may dampen the severity of a phenotype generated by DOCK6 protein depletion is an important consideration in the context of both the DOCK and Rho GTPase families, each of which have likely redundancy [55, 95]. The DOCK family contains 11 proteins that specifically regulate RAC1, CDC42 or both, leaving much scope for genetic compensation upon the pathological down-regulation of one member. Thus, vascular effects caused by loss of DOCK protein function may have been masked in genetic models due to pathway redundancy disguising the phenotype. In this case, it would be interesting to quantify the gene expression and activity of other RAC1*/*CDC42*-*specific GAPs and GEFs in vascular tissue upon DOCK protein dysregulation, to provide novel insight to additional DOCK proteins functioning within a vascular context.

Phenotypic assessment of DOCK-depleted mouse models has been largely targeted towards evaluating the impact on the nervous system (Table 1), therefore a comprehensive examination of the effect of DOCK protein loss on vascular development may be appropriate. As our understanding of the respective roles for DOCK proteins in vascular development and disease remains in its infancy, further investigation into the influence of this protein family and their regulation in vascular cell types is now warranted. Similar to DOCK proteins in non-vascular environments, it seems likely that several DOCK proteins will contribute to the migration and proliferation of critical cell types in angio- and vasculogenesis. Elucidation of the possible mechanisms of action in cytoskeleton reorganisation therefore has significant potential to provide valuable new insights into the roles of the remaining DOCK proteins and wider signalling pathways in development and maintenance of the vascular network.

## References

[1] Semenza GL (2007). Vasculogenesis, angiogenesis, and arteriogenesis: mechanisms of blood vessel formation and remodeling. J Cell Biochem.

[2] Kubis N, Levy BI (2003). Vasculogenesis and angiogenesis: molecular and cellular controls. Part 1: growth factors. Interv Neuroradiol.

[3] Blanco R, Gerhardt H (2013). VEGF and Notch in tip and stalk cell selection. Cold Spring Harb Perspect Med.

[4] Sobczak M, Chumak V, Pomorski P (2016). Interaction of myosin VI and its binding partner DOCK7 plays an important role in NGF-stimulated protrusion formation in PC12 cells. Biochim Biophys Acta.

[5] Li X, Gao X, Liu G (2008). Netrin signal transduction and the guanine nucleotide exchange factor DOCK180 in attractive signaling. Nat Neurosci.

[6] Perrault I, Hamdan FF, Rio M (2014). Mutations in DOCK7 in individuals with epileptic encephalopathy and cortical blindness. Am J Hum Genet.

[7] Miyamoto Y, Torii T, Yamamori N (2013). Akt and PP2A reciprocally regulate the guanine nucleotide exchange factor Dock6 to control axon growth of sensory neurons. Sci Signal.

[8] Ueda S, Fujimoto S, Hiramoto K (2008). Dock4 regulates dendritic development in hippocampal neurons. J Neurosci Res.

[9] Katoh H, Negishi M (2003). RhoG activates Rac1 by direct interaction with the Dock180-binding protein Elmo. Nature.

[10] Sukalo M, Tilsen F, Kayserili H (2015). DOCK6 mutations are responsible for a distinct autosomal-recessive variant of Adams-Oliver syndrome associated with brain and eye anomalies. Hum Mutat.

[11] Shi L (2013). Dock protein family in brain development and neurological disease. Commun Integr Biol.

[12] Bryan BA, D’Amore PA (2007). What tangled webs they weave: Rho-GTPase control of angiogenesis. Cell Mol Life Sci.

[13] Gadea G, Blangy A (2014). Dock-family exchange factors in cell migration and disease. Eur J Cell Biol.

[14] Cook DR, Rossman KL, Der CJ (2014). Rho guanine nucleotide exchange factors: regulators of Rho GTPase activity in development and disease. Oncogene.

[15] Cherfils J, Zeghouf M (2013). Regulation of small GTPases by GEFs, GAPs, and GDIs. Physiol Rev.

[16] Van Buul JD, Geerts D, Huveneers S (2014). Rho GAPs and GEFs: controlling switches in endothelial cell adhesion. Cell Adhes Migr.

[17] Kesavan G, Sand FW, Greiner TU (2009). Cdc42-mediated tubulogenesis controls cell specification. Cell.

[18] Barry DM, Xu K, Meadows SM (2015). Cdc42 is required for cytoskeletal support of endothelial cell adhesion during blood vessel formation in mice. Development.

[19] Laviña B, Castro M, Niaudet C (2018). Defective endothelial cell migration in the absence of Cdc42 leads to capillary-venous malformations. Development.

[20] Norden PR, Kim DJ, Barry DM (2016). Cdc42 and k-ras control endothelial tubulogenesis through apical membrane and cytoskeletal polarization: novel stimulatory roles for GTPase effectors, the small GTPases, Rac2 and Rap1b, and inhibitory influence of Arhgap31 and Rasa1. PLoS ONE.

[21] Li J, Liu Y, Jin Y (2017). Essential role of Cdc42 in cardiomyocyte proliferation and cell-cell adhesion during heart development. Dev Biol.

[22] De Smet F, Segura I, De Bock K (2009). Mechanisms of vessel branching: filopodia on endothelial tip cells lead the way. Arterioscler Thromb Vasc Biol.

[23] Fischer RS, Lam PY, Huttenlocher A, Waterman CM (2019). Filopodia and focal adhesions: An integrated system driving branching morphogenesis in neuronal pathfinding and angiogenesis. Dev Biol.

[24] Sinha S, Yang W (2008). Cellular signaling for activation of Rho GTPase Cdc42. Cell Signal.

[25] Vaggi F, Disanza A, Milanesi F (2011). The Eps8/IRSp53/VASP network differentially controls actin capping and bundling in filopodia formation. PLoS Comput Biol.

[26] Krugmann S, Jordens I, Gevaert K (2001). Cdc42 induces filopodia by promoting the formation of an IRSp53:Mena complex. Curr Biol.

[27] Gerhardt H, Ruhrberg C, Abramsson A (2004). Neuropilin-1 is required for endothelial tip cell guidance in the developing central nervous system. Dev Dyn.

[28] Fantin A, Lampropoulou A, Gestri G (2015). NRP1 regulates CDC42 activation to promote filopodia formation in endothelial tip cells. Cell Rep.

[29] Tan W, Palmby TR, Gavard J (2008). An essential role for Rac1 in endothelial cell function and vascular development. FASEB J.

[30] Caron C, Degeer J, Fournier P (2016). CdGAP/ARHGAP31, a Cdc42/Rac1 GTPase regulator, is critical for vascular development and VEGF-mediated angiogenesis. Sci Rep.

[31] Mehidi A, Rossier O, Schaks M (2019). Transient activations of Rac1 at the lamellipodium tip trigger membrane protrusion. Curr Biol.

[32] Szczepanowska J (2009). Involvement of Rac/Cdc42/PAK pathway in cytoskeletal rearrangements. Acta Biochim Pol.

[33] Wakayama Y, Fukuhara S, Ando K (2015). Cdc42 mediates Bmp-induced sprouting angiogenesis through Fmnl3-driven assembly of endothelial filopodia in zebrafish. Dev Cell.

[34] Fukui Y, Hashimoto O, Sanui T (2001). Haematopoietic cell-specific CDM family protein DOCK2 is essential for lymphocyte migration. Nature.

[35] Brugnera E, Haney L, Grimsley C (2002). Unconventional Rac-GEF activity is mediated through the Dock180-ELMO complex. Nat Cell Biol.

[36] Côté JF, Motoyama AB, Bush JA, Vuori K (2005). A novel and evolutionarily conserved Ptdlns(3,4,5)P3-binding domain is necessary for DOCK180 signalling. Nat Cell Biol.

[37] Yang J, Zhang Z, Roe SM (2009). Activation of Rho GTPases by DOCK exchange factors is mediated by a nucleotide sensor. Science.

[38] Hamel B (2010) Atypical guanine nucleotide exchange factors for Rho family GTPases: DOCK9 activation of CDC42 and SMGGDS activation of RHOA [PhD thesis]. Chapel Hill, NC: University of North Carolina at Chapel Hill. 10.17615/kmq6-qg97

[39] Miyamoto Y, Yamauchi J, Sanbe A, Tanoue A (2007). Dock6, a Dock-C subfamily guanine nucleotide exchanger, has the dual specificity for Rac1 and Cdc42 and regulates neurite outgrowth. Exp Cell Res.

[40] Kulkarni K, Yang J, Zhang Z, Barford D (2011). Multiple factors confer specific Cdc42 and Rac protein activation by dedicator of cytokinesis (DOCK) nucleotide exchange factors. J Biol Chem.

[41] Côté JF, Vuori K (2002). Identification of an evolutionary conserved superfamily of DOCK180-related proteins with guanine nucleotide exchange activity. J Cell Sci.

[42] Ruiz-Lafuente N, Alcaraz-García MJ, García-Serna AM (2015). Dock10, a Cdc42 and Rac1 GEF, induces loss of elongation, filopodia, and ruffles in cervical cancer epithelial HeLa cells. Biol Open.

[43] Ruiz-Lafuente N, Minguela A, Parrado A (2018). DOCK9 induces membrane ruffles and Rac1 activity in cancer HeLa epithelial cells. Biochem Biophys Rep.

[44] Kobialka P, Graupera M (2019). Revisiting PI3-kinase signalling in angiogenesis. Vasc Biol.

[45] Guo X, Chen SY (2017). Dedicator of cytokinesis 2 in cell signaling regulation and disease development. J Cell Physiol.

[46] Meller N, Irani-Tehrani M, Ratnikov BI (2004). The novel Cdc42 guanine nucleotide exchange factor, zizimin1, dimerizes via the Cdc42-binding CZH2 domain. J Biol Chem.

[47] Sévajol M, Reiser JB, Chouquet A (2012). The C-terminal polyproline-containing region of ELMO contributes to an increase in the life-time of the ELMO-DOCK complex. Biochimie.

[48] Chang L, Yang J, Jo CH (2020). Structure of the DOCK2 – ELMO1 complex provides insights into regulation of the auto-inhibited state. Nat Commun.

[49] Mikdache A, Fontenas L, Albadri S (2020). Elmo1 function, linked to Rac1 activity, regulates peripheral neuronal numbers and myelination in zebrafish. Cell Mol Life Sci.

[50] Toret CP, Collins C, Nelson WJ (2014). An Elmo-Dock complex locally controls Rho GTPases and actin remodeling during cadherin-mediated adhesion. J Cell Biol.

[51] Sanematsu F, Hirashima M, Laurin M (2010). DOCK180 is a Rac activator that regulates cardiovascular development by acting downstream of CXCR4. Circ Res.

[52] Kiyokawa E, Hashimoto Y, Kobayashi S (1998). Activation of Rac1 by a Crk SH3-binding protein, DOCK180. Genes Dev.

[53] Hernández-García R, Iruela-Arispe ML, Reyes-Cruz G, Vázquez-Prado J (2015). Endothelial RhoGEFs: a systematic analysis of their expression profiles in VEGF-stimulated and tumor endothelial cells. Vasc Pharmacol.

[54] Zaidel-Bar R, Kam Z, Geiger B (2005). Polarized downregulation of the paxillin-p130CAS-Rac1 pathway induced by shear flow. J Cell Sci.

[55] Epting D, Wendik B, Bennewitz K (2010). The Rac1 regulator ELMO1 controls vascular morphogenesis in zebrafish. Circ Res.

[56] Laurin M, Fradet N, Blangy A (2008). The atypical Rac activator Dock180 (Dock1) regulates myoblast fusion in vivo. Proc Natl Acad Sci USA.

[57] Katoh H, Hiramoto K, Negishi M (2006). Activation of Rac1 by RhoG regulates cell migration. J Cell Sci.

[58] Kobayashi S, Shirai T, Kiyokawa E (2001). Membrane recruitment of DOCK180 by binding to PtdIns(3,4,5)P3. Biochem J.

[59] Nishihara H, Kobayashi S, Hashimoto Y (1999). Non-adherent cell-specific expression of DOCK2, a member of the human CDM-family proteins. Biochim Biophys Acta.

[60] Cetinkaya A, Xiong JR, Vargel İ (2016). Loss-of-function mutations in ELMO2 cause intraosseous vascular malformation by impeding RAC1 signaling. Am J Hum Genet.

[61] Wang Y, Yan F, Ye Q (2016). PTP1B inhibitor promotes endothelial cell motility by activating the DOCK180/Rac1 pathway. Sci Rep.

[62] Lanahan A, Lech D, Dubrac A (2014). PTP1b is a physiologic regulator of vascular endothelial growth factor signaling in endothelial cells. Circulation.

[63] Schäker K, Bartsch S, Patry C (2015). The bipartite Rac1 guanine nucleotide exchange factor engulfment and cell motility 1/dedicator of cytokinesis 180 (Elmo1/Dock180) protects endothelial cells from apoptosis in blood vessel development. J Biol Chem.

[64] Oh J, Diaz T, Wei B (2006). TIMP-2 upregulates RECK expression via dephosphorylation of paxillin tyrosine residues 31 and 118. Oncogene.

[65] Sanders MA, Ampasala D, Basson MD (2009). DOCK5 and DOCK1 regulate Caco-2 intestinal epithelial cell spreading and migration on collagen IV. J Biol Chem.

[66] Hernández-Vásquez MN, Adame-García SR, Hamoud N (2017). Cell adhesion controlled by adhesion G protein–coupled receptor GPR124/ADGRA2 is mediated by a protein complex comprising intersectins and Elmo–Dock. J Biol Chem.

[67] Chen DY, Sun NH, Lu YP (2019). GPR124 facilitates pericyte polarization and migration by regulating the formation of filopodia during ischemic injury. Theranostics.

[68] Döring Y, Pawig L, Weber C, Noels H (2014). The CXCL12/CXCR4 chemokine ligand/receptor axis in cardiovascular disease. Front Physiol.

[69] Tachibana K, Hirota S, Iizasa H (1998). The chemokine receptor CXCR4 is essential for vascularization of the gastrointestinal tract. Nature.

[70] Strasser GA, Kaminker JS, Tessier-Lavigne M (2010). Microarray analysis of retinal endothelial tip cells identifies CXCR4 as a mediator of tip cell morphology and branching. Blood.

[71] Spuul P, Ciufici P, Veillat V (2014). Importance of RhoGTPases in formation, characteristics, and functions of invadosomes. Small GTPases.

[72] Li H, Yang L, Fu H (2013). Association between Gαi2 and ELMO1/Dock180 connects chemokine signalling with Rac activation and metastasis. Nat Commun.

[73] Jiang H, Pan F, Erickson LM (2005). Deletion of DOCK2, a regulator of the actin cytoskeleton in lymphocytes, suppresses cardiac allograft rejection. J Exp Med.

[74] Guo X, Shi N, Cui XB (2015). Dedicator of cytokinesis 2, a novel regulator for smooth muscle phenotypic modulation and vascular remodeling. Circ Res.

[75] Cao BJ, Wang XW, Zhu L (2019). Dedicator of cytokinesis 2 silencing therapy inhibits neointima formation and improves blood flow in rat vein grafts. J Mol Cell Cardiol.

[76] Dobbs K, Conde CD, Zhang SY (2015). Inherited DOCK2 deficiency in patients with early-onset invasive infections. N Engl J Med.

[77] Yan D, Li F, Hall ML (2006). An isoform of GTPase regulator DOCK4 localizes to the stereocilia in the inner ear and binds to harmonin (USH1C). J Mol Biol.

[78] Kawada K, Upadhyay G, Ferandon S (2009). Cell migration is regulated by platelet-derived growth factor receptor endocytosis. Mol Cell Biol.

[79] Kang H, Davis-Dusenbery BN, Nguyen PH (2012). Bone morphogenetic protein 4 promotes vascular smooth muscle contractility by activating microRNA-21 (miR-21), which down-regulates expression of family of dedicator of cytokinesis (DOCK) proteins. J Biol Chem.

[80] Abraham S, Scarcia M, Bagshaw RD (2015). A Rac/Cdc42 exchange factor complex promotes formation of lateral filopodia and blood vessel lumen morphogenesis. Nat Commun.

[81] Yajnik V, Paulding C, Sordella R (2003). DOCK4, a GTPase activator, is disrupted during tumorigenesis. Cell.

[82] Huang L, Chambliss KL, Gao X (2019). SR-B1 drives endothelial cell LDL transcytosis via DOCK4 to promote atherosclerosis. Nature.

[83] Stewart L, Egnuni T, Esteves F (2018). DOCK4 genetic deletion impairs vascular recovery following an ischemic event [abstract]. J Mol Cell Cardiol.

[84] Vives V, Laurin M, Cres G (2011). The Rac1 exchange factor Dock5 is essential for bone resorption by osteoclasts. J Bone Miner Res.

[85] Zhou W, Li X, Premont RT (2016). Expanding functions of GIT Arf GTPase-activating proteins, PIX Rho guanine nucleotide exchange factors and GIT-PIX complexes. J Cell Sci.

[86] Shaheen R, Faqeih E, Sunker A (2011). Recessive mutations in DOCK6, encoding the guanidine nucleotide exchange factor DOCK6, lead to abnormal actin cytoskeleton organization and Adams-Oliver syndrome. Am J Hum Genet.

[87] Meester JAN, Sukalo M, Schröder KC (2018). Elucidating the genetic architecture of Adams–Oliver syndrome in a large European cohort. Hum Mutat.

[88] Southgate L (2019). Current opinion in the molecular genetics of Adams-Oliver syndrome. Expert Opin Orphan Drugs.

[89] Lehman A, Stittrich AB, Glusman G (2014). Diffuse angiopathy in Adams-Oliver syndrome associated with truncating DOCK6 mutations. Am J Med Genet A.

[90] Jones KM, Silfvast-Kaiser A, Leake DR (2017). Adams–Oliver syndrome type 2 in association with compound heterozygous DOCK6 mutations. Pediatr Dermatol.

[91] Pisciotta L, Capra V, Accogli A (2018). Epileptic encephalopathy in Adams–Oliver syndrome associated to a new DOCK6 mutation: A peculiar behavioral phenotype. Neuropediatrics.

[92] Patel MS, Taylor GP, Bharya S (2004). Abnormal pericyte recruitment as a cause for pulmonary hypertension in Adams-Oliver syndrome. Am J Med Genet A.

[93] Kim K, Yang DK, Kim S, Kang H (2015). MIR-142-3p is a regulator of the TGFβ-mediated vascular smooth muscle cell phenotype. J Cell Biochem.

[94] Mack CP (2011). Signaling mechanisms that regulate smooth muscle cell differentiation. Arterioscler Thromb Vasc Biol.

[95] Cerikan B, Schiebel E (2019). Mechanism of cell-intrinsic adaptation to Adams-Oliver Syndrome gene DOCK6 disruption highlights ubiquitin-like modifier ISG15 as a regulator of RHO GTPases. Small GTPases.

[96] Namekata K, Guo X, Kimura A (2020). Roles of the DOCK-D family proteins in a mouse model of neuroinflammation. J Biol Chem.

[97] Brown KA, Ham AJ, Clark CN (2008). Identification of novel Smad2 and Smad3 associated proteins in response to TGF-β1. J Cell Biochem.

[98] Kashiwa A, Yoshida H, Lee S (2000). Isolation and characterization of a novel presenilin binding protein. J Neurochem.

[99] Nellist M, Burgers PC, Van Den Ouweland AMW (2005). Phosphorylation and binding partner analysis of the TSC1-TSC2 complex. Biochem Biophys Res Commun.

[100] Williams CK, Segarra M, Sierra MDLL (2008). Regulation of CXCR4 by the Notch ligand delta-like 4 in endothelial cells. Cancer Res.

[101] Carretero-Ortega J, Walsh C, Hernandez-Garcia R (2010). Phosphatidylinositol 3,4,5-triphosphate-dependent Rac exchanger 1 (P-Rex-1), a guanine nucleotide exchange factor for Rac, mediates angiogenic responses to stromal cell-derived factor-1/chemokine stromal cell derived factor-1 (SDF-1/CXCL-12) linked to Rac. Mol Pharmacol.

[102] Moore CA, Parkin CA, Bidet Y, Ingham PW (2007). A role for the Myoblast city homologues Dock1 and Dock5 and the adaptor proteins Crk and Crk-like in zebrafish myoblast fusion. Development.

[103] Cunningham RL, Herbert AL, Harty BL (2018). Mutations in dock1 disrupt early Schwann cell development. Neural Dev.

[104] Maldonado MDM, Medina JI, Velazquez L, Dharmawardhane S (2020). Targeting Rac and Cdc42 GEFs in metastatic cancer. Front Cell Dev Biol.

[105] Cimino PJ, Yang Y, Li X (2013). Ablation of the microglial protein DOCK2 reduces amyloid burden in a mouse model of Alzheimer’s disease. Exp Mol Pathol.

[106] Chen Q, Peto CA, Shelton GD (2009). Loss of modifier of cell adhesion reveals a pathway leading to axonal degeneration. J Neurosci.

[107] Helbig KL, Mroske C, Moorthy D (2017). Biallelic loss-of-function variants in DOCK3 cause muscle hypotonia, ataxia, and intellectual disability. Clin Genet.

[108] Omi N, Kiyokawa E, Matsuda M (2008). Mutation of Dock5, a member of the guanine exchange factor Dock180 superfamily, in the rupture of lens cataract mouse. Exp Eye Res.

[109] Nakamuta S, Yang YT, Wang CL (2017). Dual role for DOCK7 in tangential migration of interneuron precursors in the postnatal forebrain. J Cell Biol.

[110] Torii T, Miyamoto Y, Nagao M (2012). Knockdown of Dock7 in vivo specifically affects myelination by Schwann cells and increases myelin thickness in sciatic nerves without affecting axon thickness. Am J Mol Biol.

[111] Krishnaswamy JK, Singh A, Gowthaman U (2015). Coincidental loss of DOCK8 function in NLRP10-deficient and C3H/HeJ mice results in defective dendritic cell migration. Proc Natl Acad Sci USA.

[112] Wu J, Zhang S, Qin T (2018). IL-21 alleviates allergic asthma in DOCK8-knockout mice. Biochem Biophys Res Commun.

